# METTL Family in Health and Disease

**DOI:** 10.1186/s43556-024-00194-y

**Published:** 2024-08-19

**Authors:** Jiejie He, Fengchen Hao, Shiqi Song, Junli Zhang, Hongyu Zhou, Jun Zhang, Yan Li

**Affiliations:** 1 Department of Gynecologic Oncology, Affiliated Hospital of Qinghai University, Xining, 810000 Qinghai Province China; 2 Department of Radiology, Affiliated Hospital of Qinghai University, Xining, 810000 Qinghai Province China; 3 Department of Urology Surgery, Affiliated Hospital of Qinghai University, No. 29, Tongren Road, West of the City, Xining, 810000 Qinghai Province China; 4 Department of Gynecologic Oncology, Affiliated Hospital of Qinghai University & Affiliated Cancer Hospital of Qinghai University, No. 29, Tongren Road, West of the City, Xining, 810000 Qinghai Province China

**Keywords:** Methylation, Methyltransferase-like protein (METTL) Family, Translation, Treatment

## Abstract

Transcription, RNA splicing, RNA translation, and post-translational protein modification are fundamental processes of gene expression. Epigenetic modifications, such as DNA methylation, RNA modifications, and protein modifications, play a crucial role in regulating gene expression. The methyltransferase-like protein (METTL) family, a constituent of the 7-β-strand (7BS) methyltransferase subfamily, is broadly distributed across the cell nucleus, cytoplasm, and mitochondria. Members of the METTL family, through their S-adenosyl methionine (SAM) binding domain, can transfer methyl groups to DNA, RNA, or proteins, thereby impacting processes such as DNA replication, transcription, and mRNA translation, to participate in the maintenance of normal function or promote disease development. This review primarily examines the involvement of the METTL family in normal cell differentiation, the maintenance of mitochondrial function, and its association with tumor formation, the nervous system, and cardiovascular diseases. Notably, the METTL family is intricately linked to cellular translation, particularly in its regulation of translation factors. Members represent important molecules in disease development processes and are associated with patient immunity and tolerance to radiotherapy and chemotherapy. Moreover, future research directions could include the development of drugs or antibodies targeting its structural domains, and utilizing nanomaterials to carry miRNA corresponding to METTL family mRNA. Additionally, the precise mechanisms underlying the interactions between the METTL family and cellular translation factors remain to be clarified.

## Introduction

**Fig. 1:**
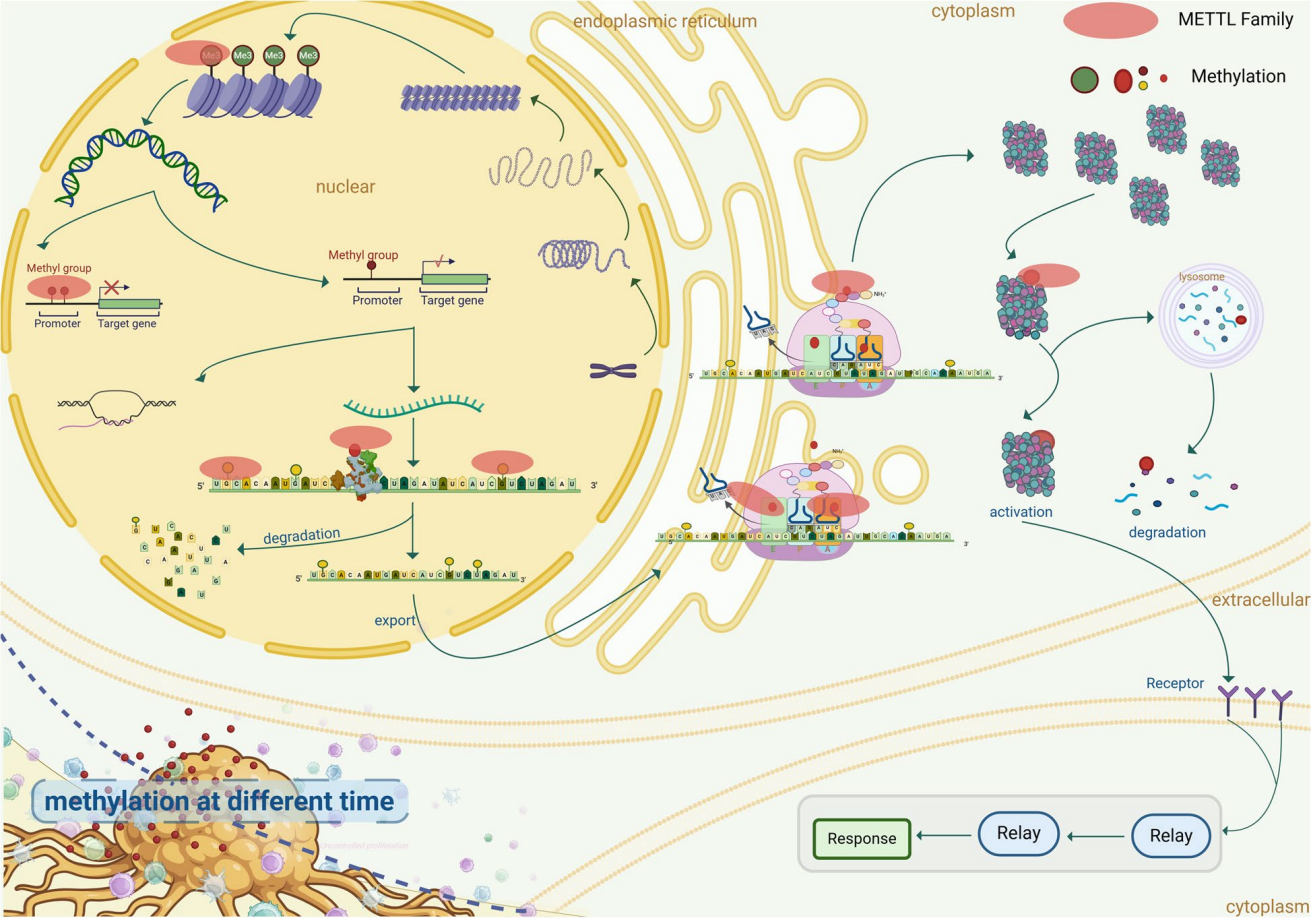
Modifications Involved in The Process of Gene Expression: From Transcription to Protein Activation and Depolymerization. Numerous processes are involved in gene expression. Histone modifications facilitate DNA transcription. DNA, in turn, undergoes modifications within proteins. mRNA modification is employed for nuclear and cytoplasmic localization. Once mRNA is in place, its modifications aid translation and facilitate the folding of the synthesized polypeptide chain, promoting protein activation and depolymerization

The widely accepted concept in eukaryotic cells is that genetic information flows from DNA to RNA and then to proteins. Proteins are the primary mediators of life functions, and their types and abundance largely determine the phenotype of cells and individuals. DNA, RNA, and proteins undergo modifications that change their structure and activity, thereby altering the process of gene expression (Fig. 1). These modifications make the simple expression of DNA and RNA more complex, no longer relying solely on simple recognition for transcription and translation. DNA methylation changes the DNA conformation, reducing its stability and causing chromatin inactivation and DNA silencing, leading to restricted embryonic development or the occurrence of tumors and diseases [1]. RNA modifications [2, 3], by affecting RNA stability and translation efficiency, regulate gene expression [4]. Methylation and acetylation modifications not only catalyze protein activity but also indirectly change the structure and function of RNA or DNA, thereby altering the organism's pathophysiological processes [5–7].

Methylation modifications affect the structure and function of DNA and RNA. In mammals, there is an average of one m6A modification for every 700–800 nucleotides, and a single mRNA contains an average of 3–5 m6A modifications, which participate in mRNA maturation, degradation, and translation. Methylation modifications on ribosomal RNA (rRNA) or ribosomal proteins lead to changes in the cell's translation rate, as well as in the length and variety of polypeptide chains, resulting in alterations in cellular biological functions. Histone modifications regulate the extent of chromatin relaxation, affecting the binding of transcription factors and promoting transcriptional activity of RNA polymerase. Different types or levels of modifications can induce protein aggregation or disassembly, or alter the effects of downstream DNA, RNA, or proteins. Methylation modifications are widely present in DNA, RNA, and proteins, and are currently the most researched form of modification, compared to other types of modifications. In addition, methylations are not confined to the cytoplasm and nucleus; they also play a crucial role in cellular growth and reproduction processes within mitochondria.

**Fig. 2:**
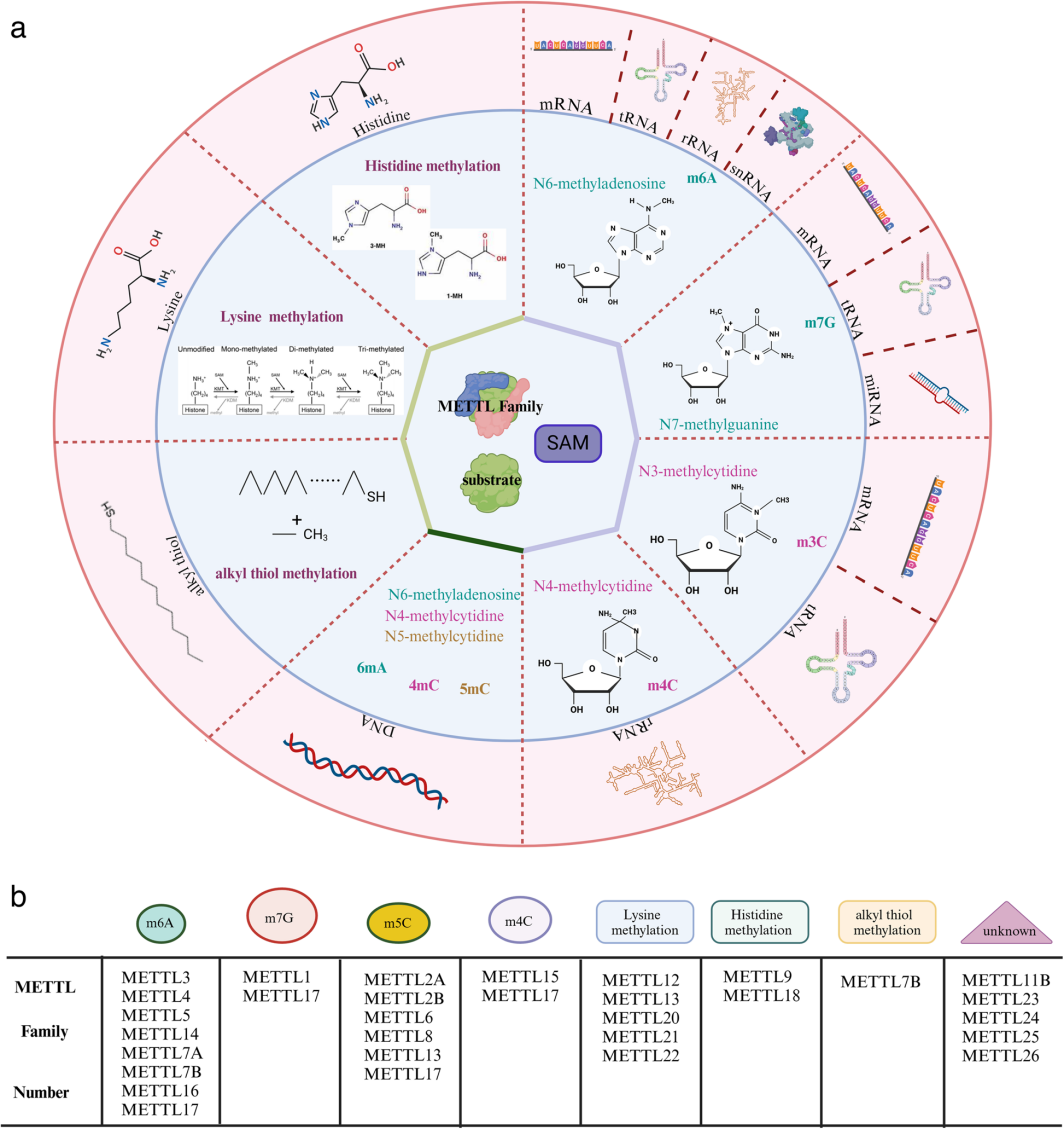
Various Methylation Modifications Produced by the METTL Family. **a** The METTL family belongs to the 7BS methyltransferase subfamily, which possesses a SAM binding domain and has the ability to catalyze various types of methylation modifications on DNA, RNA, and proteins. Introduce methyl groups to various ligands, such as DNA, RNA, and proteins, to induce different types of methylation modifications. **b** The presence of different types of modifications in METTL family members

Methyltransferase (MTase) is a pivotal enzyme responsible for catalyzing methylation and is ubiquitously distributed throughout the human body. The METTL family, a subset of the 7BS methyltransferase, comprises an SAM binding domain, leading to various types of methylation in DNA, RNA, and proteins located in the nucleus, cytoplasm, and mitochondria (Fig. 2a, b). This has implications for DNA replication, transcription, mRNA translation, histone function, chromosome structure [8–11], and is associated with drug resistance [12]. As part of the "writer" category, the METTL family transfers m6A to mRNA, with "reader" proteins identifying and binding to target RNA, thus recruiting translation initiation factors to commence translation. Additionally, certain members methylate histone amino acids and translation regulatory factors, thereby influencing chromatin remodeling, mRNA translation initiation, and elongation. Both within and outside the mitochondria, the METTL family impacts the translation process and mitochondrial function through the methylation of mitochondrial protein-related genes or direct modification of membrane proteins. Notably, the modification of translation factors by the METTL family stands out among its effects on other protein modifications.

This review commences by delineating the structural and functional attributes of various members within the METTL family, with a concise discussion on their architecture, enzymatic activity, and ligand specificity. Subsequently, it explicates the involvement of the METTL family in both physiological and pathological conditions, with specific emphasis on its role in cellular differentiation, maintenance of mitochondrial functionality, as well as its implication in malignancies, neurodegenerative disorders, and cardiovascular ailments. Finally, it underscores the potential of the METTL family as prognostic markers and delineates prospective research directions and challenges for therapies. The primary aim of this review is to furnish insights that could potentially advance disease management in the future.

## METTL family: structure and function

### Family members

#### METTL1

Located on chromosome 12q14.1 [13], METTL1 consists of 276 amino acids and can form 8 α-helices and 7 β-sheets. Through its N-terminal SAM binding domain and C-terminal RNA binding domain, METTL1 catalyzes the transfer of m7G to tRNA [14, 15], mRNA [16], miRNA [17, 18], and lncRNA [19], introducing a positive charge to RNA and subsequently impacting cellular biological behaviors [20, 21], immune microenvironment, autophagy, chemotherapy sensitivity, and immunotherapy. METTL1 plays a vital role in maintaining the normal structure and function of the human body and is associated with physiological processes such as DNA repair [22, 23], cardiac fibroblast regeneration [24, 25], and neurogenesis [26–28]. Specific knockout of METTL1 in fibroblasts has been shown to mitigate myocardial infarction-induced heart failure and myocardial fibrosis. Moreover, METTL1 is responsible for maintaining homeostasis, including PTH and FGF23 levels, thereby preventing the toxicity of 1,25(OH)(2)D(3) and regulating adaptive vitamin D metabolism and mineral [29, 30]. An imbalance of METTL1 has been linked to various diseases. Reduced levels of m7G modification on Sptbn2 mRNA due to METTL1 deficiency inhibit stability and translation, leading to impaired hippocampal neurogenesis and spatial memory in adult mice, ultimately contributing to Alzheimer's disease(AD) [31]. Additionally, METTL1, in conjunction with WDR4, acts as a scaffold, binding to the variable loop of tRNA through its αC and α6 helices and catalyzing the methylation modification of tRNA G46 to stabilize tRNA [32, 33]. Notably, an imbalance of the METTL1/WDR4 complex has been associated with the development and occurrence of various types of tumors [16, 34]. With its dual role as an oncogene and a tumor suppressor gene, downregulation of tRNA m7G methylation by METTL1 has been shown to promote the proliferation and angiogenesis of human-induced pluripotent stem cells in nude mice, leading to the formation of teratomas [35].

#### METTL3/METTL14

METTL3 and METTL14, as members of the METTL family, are extensively studied for their roles in methylation modification, both individually and in combination [36–40]. METTL3 contains a methyltransferase domain (MTD) and a zinc finger domain (ZFD), with MTD responsible for binding with SAM and ZFD responsible for the specific recognition of the RNA GGACU sequence [41]. METTL14 has its own methyltransferase domain with a different catalytic sequence [42], but it may have lost its catalytic activity [43]. Often working together [44], the METTL3/METTL14 complex plays an oncogenic role in various cancers, including breast [45], kidney [46, 47], and liver cancer [48, 49], but has an anti-cancer effect in neuroblastoma [50]. Preliminary studies have explored cancer treatments targeting the METTL3-METTL14 complex [51].

Both METTL3 and METTL14 play crucial roles in DNA methylation [52, 53], DNA damage repair [54, 55], and maintenance of RNA stability [55–57]. Specifically, METTL3 interacts with the RNA helicase DDX21 when nascent RNA forms an R-loop by hybridizing with DNA, subsequently affecting DNA transcription [58]. Additionally, in collaboration with ARID1A, the R-loop recruits METTL3 and METTL14, leading to m6A methylation modification of RNA on the R-loop, which in turn assists in driving RNA degradation and promoting DNA end resection at the DSB, ultimately ensuring genome stability [59]. Moreover, high levels of methylation caused by METTL3 on the spliceosome can lead to abnormal splicing [60].

Furthermore, METTL3/METTL14 is closely linked to organelle function and disease development. METTL3 induces mitochondrial fission, promoting the progression of glioblastoma [61], while silencing METTL14 alleviates liver injury caused by mitochondrial homeostasis disorder in non-alcoholic fatty liver disease [62]. In the context of human cytomegalovirus (HCMV) infection, m6A modification-mediated mRNA decay and R-loop-dependent transcription termination significantly reduce UCHL1 expression, leading to FDXR ubiquitination and degradation. FDXR deficiency can cause mitochondrial iron overload, resulting in AIM2 inflammasome activation and endothelial damage. Notably, METTL3 inhibitors restore UCHL1 expression and inhibit endothelial cell inflammation and injury [63]. This modification plays a crucial role in maintaining organelle stability. Additionally, endoplasmic reticulum stress significantly increases m6A modification levels through XBP1-dependent transcriptional upregulation of METTL3/METTL14, which in turn regulates the mRNA stability of endoplasmic reticulum autophagy regulators CALCOCO1 and p62, thereby promoting endoplasmic reticulum autophagy [64].

#### METTL4

METTL4, even though belonging to the same MT-70A family as METTL3/METTL14, shares 65% specificity in recognizing Am and transferring m6A with METTL3/METTL14 [65]. It is situated on chromosome 18p11.32, found in the cytoplasm, mitochondrial matrix, and cell nucleus, and it is highly conserved throughout evolution [66]. Studies indicate that METTL4 specifically modifies the 30th Am (2'-O-methyladenosine) of the U2 snRNA using the HMAGKD sequence as the target [66, 67], thereby affecting mRNA splicing [68]. In human cells, the absence of METTL4 impacts the m6Am characteristics of splicing events in U2 snRNA, leading to defects in 3' splicing sites and an increased number of exons in mRNA. Additionally, research has shown that METTL4 is crucial for the 6mA modification of DNA in mitochondria and plays a role in regulating mRNA m6A modification. Furthermore, the mutation of the direct homologous gene MTA1 of METTL4 in the rice blast fungus amplifies the amount of MoATG8 mRNA, resulting in autophagy disorders in plant pathogenic fungi [69].

6mA is a significant epigenetic mark in eukaryotes, playing a regulatory role in biological processes such as DNA transcription, transposon activation, and stress response [70]. In animals and plants, the level of 6mA is high only in organellar DNA, while it is very low in nuclear DNA [71]. The DNA 6mA modification by METTL4 in cells regulates the INSR signaling pathway, which promotes sugar uptake and lipid generation in the terminal differentiation stage, influencing the differentiation of immature fat cells [72]. Changes in environmental hypoxia mediated by METTL4 lead to the enrichment of 6mA in mammalian tumor cells, activating multiple metastasis-inducing genes and resulting in tumor metastasis [73]. Besides its critical role in the cytoplasm, METTL4 mediates mt-DNA 6mA modification, which leads to weakened transcription and reduced copy number of mt-DNA. Knockdown of METTL4 causes a decrease in the level of mt-DNA 6mA modification, inhibiting the binding of mitochondrial transcription factor A (TFAM) to DNA and affecting translation efficiency, while the levels of m6A and m6Am methylation on rRNA, mRNA, and snRNA remain unchanged [74]. METTL4 promotes gene expression and also inhibits normal cell function through related mechanisms. Studies in mouse models have demonstrated that drugs targeting mt-DNA can slow tumor development without suppressing the growth of healthy cells [75], as METTL4 can cause cell arrest in the G1 phase or lead to chromosome alignment disorders, thereby controlling the cell cycle process [76].

#### METTL5

METTL5 is situated on chromosome 2q31.1 and exhibits SAM binding activity as well as rRNA m6A methyltransferase activity, with a specific binding affinity to the 18S rRNA UAACA sequence at positions [77–79]. Deletion of METTL5 leads to an 80% reduction in polysome formation [80], resulting in alterations to their quantity and conformation, ultimately causing a deceleration in mRNA translation and protein synthesis rates. In cellular contexts, TRMT112 and MTase interact with each other, and deletion of TRMT112 decreases the levels of METTL5 protein. The heterodimeric complex formed by these two proteins promotes the methylation modification of adenine at position 1832 on 18S rRNA [81–83]. This complex modulates the m6A modification at the 5' end of c-MYC mRNA and the coding DNA sequence region (near the 5'UTR), thus regulating c-MYC expression and advancing the progression of pancreatic cancer [82].

Moreover, METTL5 plays a role in the regulation of cell differentiation. In mouse embryonic stem cells (mESCs), decreased levels of KIF4, Nanog, Sox2, and Rex1/Zfp42 mRNA and proteins are observed in response to reduced METTL5 expression, resulting in an overall decline in cell translation levels [84, 85]. Deletion of METTL5 reduces the m6A levels formed by 18S rRNA A1832, leading to an overall decrease in translation rate, spontaneous loss of pluripotency, and impaired differentiation potential [86].

#### METTL7A/B

METTL7A and its homolog METTL7B are thiol methyltransferases located on chromosome 12q13.12 and 12q13.2, respectively. Both enzymes catalyze the transfer of SAM to receptor substrates containing alkyl and phenolic thiols. They are both situated in the endoplasmic reticulum. METTL7A is considered a tumor suppressor gene with multiple editing sites at its 3' end, and its activity is regulated by RNA editing, dsRNA binding, and inhibition by ADARs [87]. In adipocytes, METTL7A utilizes its methyltransferase activity to m6A-modify long non-coding RNAS (lncRNAs) LOC606724 and SNHG1, which increases their stability for release into exosomes. These modified lncRNAs have been shown to induce apoptosis in multiple myeloma cells and confer chemotherapy resistance [88]. The RhoGTPase family members RhoBTB1 and RhoBTB2 regulate the expression of METTL7B and METTL7A, respectively. Mutations or silencing of RhoBTB1 and RhoBTB2 in various epithelial cancers have been linked to compromised Golgi apparatus integrity, leading to Golgi fragmentation and promoting invasion of breast cancer cells [89]. The exact relationship between the m6A methylation function of METTL7A/B and the Golgi apparatus is not yet clear. Additionally, the specific mechanisms by which METTL7B takes advantage of the Golgi apparatus to regulate the secretion of translated proteins require further investigation [90]. It is also unclear whether both METTL7A and METTL7B have a synergistic effect on the secretion of the Golgi apparatus after m6A modification of the same downstream molecules, such as lncRNA LOC606724 and lncRNA SNHG1.

#### METTL16

The m6A methyltransferase METTL16 is situated on chromosome 17p13.3 and comprises 562 amino acids, including a methyltransferase domain and two RNA binding domains [91–93]. METTL16 is involved in the methylation of rRNA, as well as mRNA, lncRNA, and snRNA [94, 95]. The m6A-modified U6 snRNA enhances the translation of mRNA 5' exons, contributing to increased protein sequence diversity and a substantial rise in intron numbers during eukaryotic evolution [96, 97]. However, m6A modification on pre-mRNA is frequently located at its 3’ end (AG), affecting mRNA splicing and impeding protein synthesis [98, 99]. MAT2A is an SAM synthase found in most human cells, with its 3' end containing six conserved hairpin structures (hp1-6) that can be methylated by METTL16. Through interactions with hp1 and hp2-6, METTL16 post-transcriptionally regulates MAT2A, leading to the stabilization of MAT2A mRNA and increased translation.

#### METTL13

The METTL13 gene, situated on chromosome 1q24.3 [100], encodes a protein containing two methyl-binding domains, MT13-N and MT13-C [101]. Electron microscopy has revealed the widespread presence of METTL13 in the cytoplasm, mitochondria, and nuclei of HeLa and ES cells [102]. While METTL13 is generally highly expressed in most human cancer tissues, its expression is weak in normal brain, testicular, and liver tissues [103]. It has been implicated in the development of various diseases. In cancer patients, levels of METTL13 in plasma are significantly elevated compared to those in normal individuals [102]. Furthermore, METTL13 is identified as a novel oncogene, demonstrating anti-apoptotic properties when cleaved by Caspase-3 in apoptotic cancer cells [103]. Its 3' end can be targeted by miR-16 to suppress its expression [104, 105]. Moreover, in multiple cancer cell types [106, 107], METTL13 enhances malignant cellular behaviors such as proliferation, migration, and invasion [106]. Immunogenic METTL13 triggers an immune response in mice, with the antibodies produced not harming normal tissues [102]. Additionally, miR-16 can bind to the 3' end of METTL13 to decrease its expression. Indeed, METTL13 functions as a lysine-specific methyltransferase, with MT13-N specifically methylating Lys55 of eukaryotic translation elongation factor 1A (eEF1A), a reaction that is highly specific and widespread in mammalian cells and tissues [108, 109], leading to reduced efficiency of amino acid synthesis and slowing down protein production.

#### METTL15

The gene encoding the m4C methyltransferase METTL15 is located on chromosome 11p14.1 and plays a critical role in methylating the 839th base C of the 12S mt-rRNA [110, 111]. This modification promotes the folding of the 12S rRNA, impacts the assembly of mitochondrial small ribosomal subunits, and influences mitochondrial protein translation and respiration. The m4C modification level in rRNA also affects mRNA translation efficiency. Depleting METTL15 results in elevated intracellular reactive oxygen species and reduced membrane potential, subsequently causing alterations in cellular metabolism. In addition to its interaction with 12S rRNA, METTL15 promotes cellular lactate secretion, including the lactylation of histone H4K12 and H3K9, through its interaction with hsRBFA and other mitochondrial small subunits [112, 113]. Furthermore, RBFA aids in recruiting the methyltransferase TFB1M to the unfolded H45 rRNA, which in turn accelerates rRNA maturation by promoting the binding of METTL15 to rRNA, consequently altering the conformation of RBFA [114]. Knocking down miR-374a-5p or ESCO2 can reverse the malignant cellular behaviors induced by METTL15, indicating its potential anti-cancer effects [115].

#### METTL17

METTL17 is significantly involved in stabilizing the methylation enzyme of mitochondrial ribosomal RNA. It functions alongside mitochondrial 12S rRNA and small ribosomal subunits to facilitate the assembly of mitochondrial ribosomes, thus promoting normal mESC differentiation [116]. Serving as a checkpoint for Fe-S clusters, METTL17 plays a crucial role in the translation of oxidative phosphorylation (OXPHOS) proteins containing these clusters [117]. Reduced expression of Fe-S cluster biosynthesis and delivery factors can adversely affect mitochondrial stability [118]. Conversely, upregulation of METTL17 can rectify deficiencies in cellular mitochondrial translation and bioenergy and inhibit cell growth resulting from mitochondrial protein depletion. Conversely, decreasing METTL17 expression can disrupt mitochondrial function and energy metabolism, while intensifying cellular and mitochondrial lipid peroxidation and ROS levels. Suppression of METTL17 expression leads to compromised translation of mitochondrial protein-coding genes through the inhibition of mt-RNA methylation modification. Moreover, its interaction with protein partners is pivotal for mitochondrial gene expression, and its decreased expression can induce ferroptosis in colorectal cancer cells, thereby inhibiting cell proliferation [119]. Consequently, METTL17 is intricately linked to mitochondrial translation.

#### METTL8, METTL2A/B and METTL6

METTL8 exhibits both methyltransferase and histone acetyltransferase activities. In mammals, it is the first confirmed methyltransferase capable of adding m3C to mRNA and functioning as a methyltransferase for mt-tRNA [120–122]. Alternative splicing of METTL8 mRNA leads to the formation of different isoforms, including METTL8-iso1 (localized in mitochondria) and METTL8-iso4 (distributed in the nucleolus) [123]. METTL8-iso1 is linked to the respiratory chain and regulates cellular energy synthesis or breakdown by balancing complex I in the mitochondrial respiratory chain, which consists of mitochondrial ND1 and ND6, thereby influencing cell metabolism. As a mitochondrial SAM-dependent methyltransferase, METTL8 also facilitates the modification of m3C at the 32nd position on the anticodon loops of mitochondrial tRNA(Ser)(UCN) and tRNA(Thr) [122].

On chromosome 17q23.2, METTL2A is generally expressed at lower levels in most tissues, while METTL2B, located on chromosome 7q32.1, shows higher expression levels in the liver and skeletal muscle tissues [124]. Both METTL2A and METTL2B are similar and involved in tRNA m3C methylation, maintaining anticodon base repair and folding, and regulating gene expression, translation, cellular homeostasis, and tumor growth [125]. Deletion of METTL2 leads to a 30–40% decrease in tRNA m3C methylation, mainly targeting the 32nd position on the tRNA base [120]. There is an overlap in the modification sites of METTL2 with those of METTL8, and further research is needed to establish their coordinated actions.

Situated on chromosome 3p25.1, METTL6 acts as an m3C methyltransferase [120, 126], methylating the "UGA" encoding SER-tRNA. METTL16 acts on Soga1 mRNA, reducing chromosomal stability and causing a delay in cell mitosis [127]. Additionally, METTL6 promotes cell proliferation, migration, and invasion [128]. The absence of METTL16 affects the cellular translation process and protein homeostasis, resulting in defects in pluripotency potential [129], altered glucose homeostasis, and metabolic imbalance in mESCs [130].

#### Others

METTL18 methylates the 60S ribosomal protein L3 (RPL3) at the τ-N position of His245, effectively retarding the ribosomal movement on Tyr codons. This deceleration provides additional time for the proper folding of the peptide chain [131, 132], facilitating the formation of functional domains. In addition to methylating lyso-368/395 within citrate synthase (CS) in the mitochondrial matrix [133, 134], METTL12 also regulates the channel proteins in the respiratory chain, thereby influencing protein–protein interactions during the metabolic process. Within HEK293T cells, METTL20 forms complexes with Lys-199, Lys-200, Lys-202, and Lys-203 in ETFβ [135, 136], facilitating trimethylation of ETFβ. This process links the mobile electron carrier, ETF, to the membrane-bound ubiquinone pool, thereby influencing metabolic pathways associated with fatty acid oxidation and one-carbon metabolism dehydrogenases [137]. Furthermore, the METTL21 protein (A-E) catalyzes lysine methylation of molecular chaperones and eEF1A, which is closely associated with human health and disease [138].

### Discussion on their structure, enzymatic activity, and substrate specificity

**Fig. 3:**
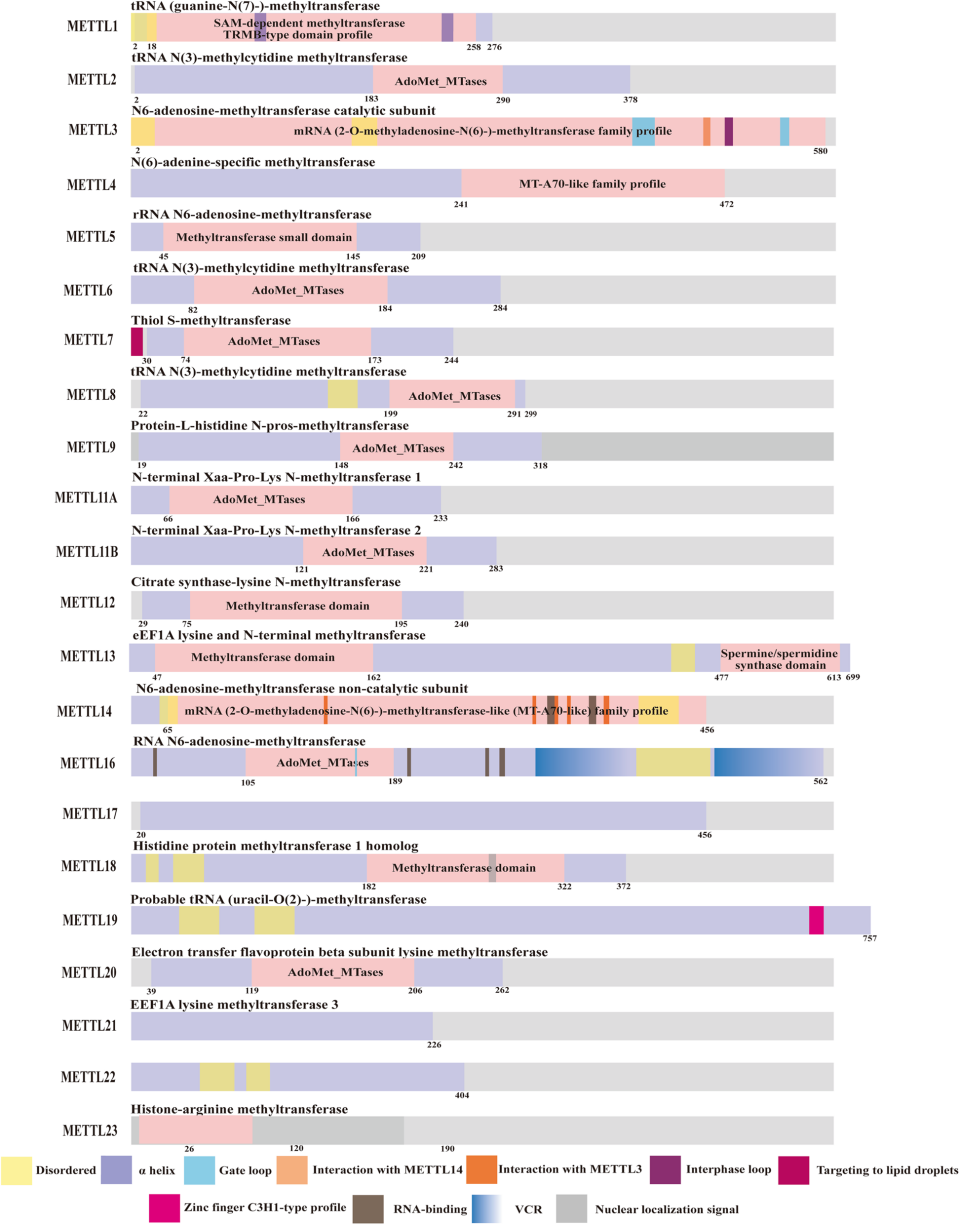
A comprehensive look at the structure of the METTL family members. The protein structures have been determined for a subset of METTL family members, based on data obtained from the Uniprot Protein Database. The figure presents the structural findings from existing studies on the family members. It is observed that some members of the family share the same functional domain structure and are of roughly similar size

The 7BS methyltransferase family and SET domain methyltransferases, including Su(var) 3–9, enhancer of zeste, and Trithorax, represent two major families of methyltransferases. The presence of a conserved SAM binding domain classifies members of the METTL family into the 7BS methyltransferase subfamily. In contrast to the SET domain family, which exclusively methylates lysine residues in proteins, METTL family members exhibit broader methylating capabilities, extending to various amino acids in proteins, such as lysine and arginine, as well as nucleic acids and other small molecule metabolites [139]. While some METTL family members share similar functional domains (Fig. 3), they may exert different effects on the same ligand. For instance, METTL6, METTL7, and METTL8 all possess the Ado-MTase domain, however, they lead to distinct methylation modifications, with METTL6 forming m6A modifications, while METTL7 and METTL8 form m3C. METTL family demonstrates substrate specificity, with some primarily responsible for methylating mRNA, tRNA, microRNA, rRNA, and mtRNA, and others primarily regulating the formation of methylated nuclear or mitochondrial DNA as well as cytoplasmic proteins. This differential action could be attributed to other molecular structural domains exhibiting a higher affinity for specific substrates or altering the conformation of the methyl-binding domain. Moreover, it may be related to the varied concentration of METTL family members in different cellular locations, resulting in competitive substrate binding. Additionally, METTL family members can form complexes with each other or with other proteins (METTL13-METTL11A, METTL2A/B or METTL7A/B, METTL1-WDR4, METTL2-DALRD3, METTL5-TRMT112), consequently impacting the activity of acting molecules and methylation modifications. The formation of these complexes may lead to changes in molecular structure, resulting in different outcomes compared to individual actions, or may influence the localization of molecules within the cell, potentially increasing their concentration in the nucleus, cytoplasm, or within specific organelles, such as the ribosome or mitochondria, allowing them to compete with other family members.

While current research mainly focuses on single modifications, the exploration of dual modifications of substrates by individual or complexed METTL family members could be a future research direction. In the context of malignant transformation of bladder epithelial cells, the methyltransferase METTL3, which mediates programmable m6A modifications, enhances the translation of the oncogene Trophoblast 2 (TROP2) mRNA. The m7G methyltransferase METTL1 boosts the translation of TROP2 by mediating m7G modifications of certain tRNAs [140]. METTL13 also exhibits a dual modification effect on eEF1A, with lysine 55 methylation potentially exerting a greater impact on eEF1A translation.

### Functions of METTL family members in epigenetic regulation and RNA processing

The METTL family is involved in the regulation of RNA translation, including translation factors, through RNA methylation modification. It also facilitates the involvement of translation factors in translation activity through methylation modification or serves as a link to promote intracellular translation using translation factors. In neurons, METTL1 places m7G at the 5' end of SPTBN2, thus enhancing mRNA stability and translation speed, leading to the proliferation of neurons and glial cells, ultimately contributing to the pathogenesis of AD [31]. The m7G cap modification is essential in regulating the RNA life cycle, stable transcripts, and translation [141]. Additionally, m7G has been observed not only at the 5' end of mRNA but also in the internal region of mRNA [142]. In acute myeloid leukemia (AML), the knockout of METTL1 reduces the m7G methylation level of mRNA and tRNA, resulting in a significant decrease in protein synthesis [15]. Furthermore, rRNA plays a critical role in translation, as knocking down METTL5 inhibits the formation of 18S rRNA m6A modification, subsequently hindering ribosome synthesis and suppressing mRNA modification in the G-quadruplex of the TGF-β pathway, leading to inhibited mRNA translation in cholangiocarcinoma [79]. Moreover, bioinformatics analysis revealed that in MCT-PAH rats, there was an upregulation of METTL3, WTAP, and YTHDF1, and the eIF2α mRNA with common sequences of m6A modification was also upregulated, thereby promoting the occurrence and development of pulmonary arterial hypertension [143]. Cell experiments conducted after the transfection of lung adenocarcinoma cells with shMETTL3 exhibited inhibited m6A modification and a significant reduction in eIF expression [144]. METTL13 methylates eEF1A Lysine55, thereby promoting mRNA translation. Furthermore, METTL16, in cases not dependent on its m6A modification of mRNA, promotes its own and mRNA 5' cap structure binding, and nuclear ribosome translation level through its Mtase domain complex with eIF3a/b.

**Fig. 4:**
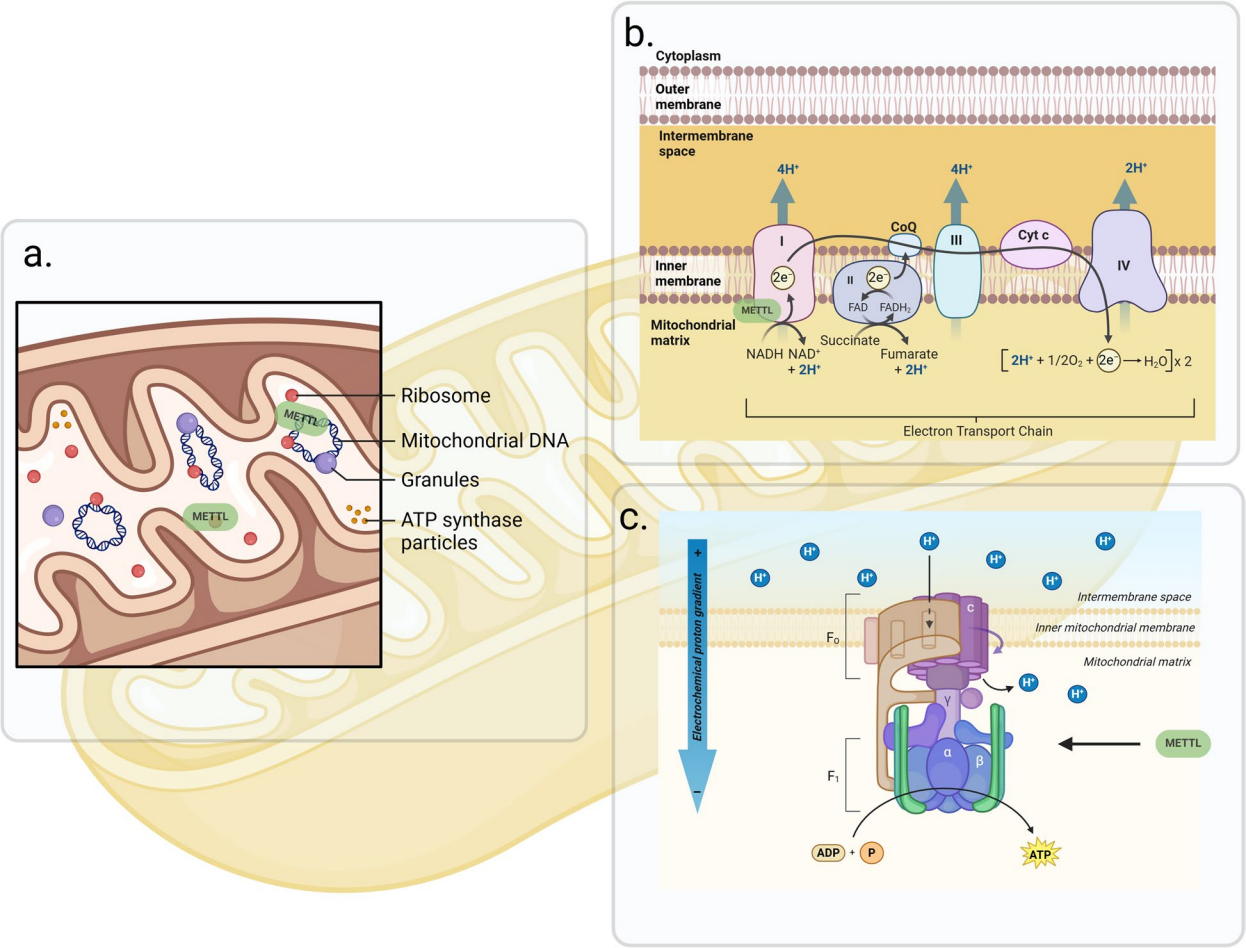
The Role of the METTL Family in Mitochondria. Inside and outside the mitochondria, the METTL family methylates mitochondrial protein genes, thereby directly influencing the translation process, and disturbs the function of the proteins within the mitochondria

In addition to the impact of RNA modification on translation, gene expression is regulated not only by the level of ribosomal components or related molecular modifications, but is also influenced by mitochondria (Fig. 4). The methylation level of related molecules on mitochondria ultimately affects the translation function of cytoplasmic ribosomes. The respiratory chain complex I formed by mitochondrial ND1 and ND6 is regulated by METTL8-iso1, thus controlling intracellular energy synthesis or decomposition processes via the formation of GTP-driven tRNA translocation [123]. METTL12 controls the channels on the mitochondrial membrane, affecting the interaction of protein–protein in the metabolic process, and the membrane channels form important pathways for the formation of intra- and extra-membrane ion gradients [133, 134]. The decreased level of mt-DNA 6mA modification leads to reduced protein synthesis and affects mitochondrial function [74]. The stable state of mitochondria provides a sufficient energy source for intracellular translation activity.

## Role of METTL family in health and disease

To date, researchers have predominantly concentrated on the atypical expression of METTL family members, the onset, and progression of tumors, and have investigated mechanisms associated with malignant biological behaviors in the majority of tumors. Nevertheless, the regular expression levels of the METTL family are essential for preserving normal cellular functions and can enhance cell differentiation and maintain stable mitochondrial function (Table 1). Moreover, apart from its involvement in tumors, its dysregulation is also noteworthy in diseases linked to the nervous and cardiovascular systems.

**Table 1:** Role of METTL Family in Health

	**Regulators**	**Targets**	**function**	**References**
**Differentiation**	METTL1	tRNA(m7G)	METTL1-mediated m7G modification in the regulation of hiPSC proliferation and angiogenesis	[35]
		MAPK/ERK pathway	hiPSC differentiation into EPCs in vitro; phenotype switching of vascular smooth muscle cells under co-culture system	[145]
		–	osteogenic differentiation of human ESCs	[146]
	METTL4	DNA(6mA)	adipogenesis, a positive correlation between the density of promoter 6 mA and gene expression level	[72]
	METTL5	rRNA(m6A)	a decrease in global translation rate, spontaneous loss of pluripotency, and compromised differentiation potential	[86]
		rRNA(m6A)	neural development and brain function	[147]
	METTL6	tRNA(m3C)	changes in ribosome occupancy and RNA levels, as well as impaired pluripotency	[130]
	METTL8	Mapkbp1(mRNA)	ESCs differentiation	[148]
	METTL16	mybl2b (m6A)	G1/S cell cycle arrest	[149]
		eIF3a	liver cancer stem cell self-renewal via controlling ribosome biogenesis and mRNA translation	[150]
		–	a negative correlation between METTL16 and EMT	[151]
		MCP1 (m6A)	MSCs transfected with METTL16 siRNA showing greater ability to recruit monocytes	[152]
**Mitochondria**	METTL4	mt-DNA(6mA)	mitochondrial stress response	[74]
		Nrf2(m6A)	mitochondrial transcription and ferroptosis	[153]
	METTL8	mt-tRNA(m3C)	associate with mitochondrial ribosomes for translation	[121]
		mt-tRNA(m3C)	a functional connection between mitochondrial tRNA modification and charging	[154]
		mt-tRNA(m3C)	mitochondrial protein expression and neural stem cell maintenance in human forebrain cortical organoids	[155]
		mt-tRNA(m3C)	facilitate the optimal composition and function of the mitochondrial respiratory chain	[122]
	METTL12	Lys368 methylation	control substrate channel, or influence protein–protein interactions in the metabolon	[133]
		CS (Lys395 methylation)	CS activity diminished by methylation	[134]
	METTL15	mt-rRNA	impaired translation of mitochondrial protein-coding mRNAs and decreased mitochondrial respiration capacity	[110]
		mt-rRNA	a reduction of the mitochondrial de novo protein synthesis and decreased steady-state levels of protein components of the oxidative phosphorylation system	[111]
		mt-rRNA	perturb the composition of mitochondrial protein biosynthesis machinery	[113]
	METTL16	MAT2A(m6A)	oxidative stress	[156]
	METTL17	mt-rRNA methylation	mitochondrial ribosome assembly	[116]
		–	bind to the mitoribosomal small subunit during late assembly and harbor a previously unrecognized [Fe_4_S_4_]_2_^+^ cluster required for its stability	[117]
		mt-rRNA methylation	METTL17-mediated defense mechanism for cell survival and ferroptosis	[119]
		mt-rRNA methylation	significant changes in mitochondrial oxidative phosphorylation and cellular metabolome	[157]
	METTL20	ETFβ ((Lys199 and Lys202 methylation)	the oxidation of fatty acids in mitochondria and the passage of electrons	[135]
		ETFβ ((Lys200 and Lys203 methylation)	regulate cellular metabolism through modulating the interaction between its substrate ETFβ and dehydrogenases	[136]
		ETFβ ((Lys193 and Lys196 methylation)	electron transferring ability	[158]
**Others**	METTL3	mTOR	VSMC proliferation and autophagy	[159]
	METTL4	Col17a1, Itgβ4 and Itgα6(m6A)	the maintenance of epidermal homeostasis	[160]
	METTL13	–	prevent the deafness caused by GAB1 gene mutations	[161]
		–	postpartum emotional syndrome	[162]
		Ezrin, Rab8a, and Septin2	abdominal testicular migration	[163]

### Role of METTL family in health

#### METTL family and differentiation

The METTL family is closely associated with stem cell differentiation, and inhibiting it can block embryonic differentiation. Knocking down METTL1 in stem cells reduces the level of m7G modification on tRNA or mRNA, as well as the translation efficiency of stem cell transcription factors OCT4, SOX2, and Nanog, leading to mesoderm differentiation, including new blood vessel formation, while inhibiting neural ectoderm differentiation. Furthermore, it can accelerate the conversion of human induced pluripotent stem cells into endothelial progenitor cells (EPCs), as well as the proliferation and phenotypic transition of vascular smooth muscle cells [145]. In METTL16 knockout cells, the level of pluripotency factor RNA is reduced, including RNA levels of pluripotency-related transcription factors (such as Zfp42 (Rex1), Klf4, Esrrb, Tbx3, and Dppa3) and signal pathway genes (such as Lifr), leading to increased stem cell differentiation [130]. However, the loss of METTL5 in stem cells leads to the spontaneous loss of overall pluripotency and impaired differentiation potential [86], with the deletion of METTL5 mediating defects in m6A modification of 18S rRNA in stem cell neurodifferentiation, regulating key functions in neural development and brain function [147]. Some members of the METTL family only regulate the translation of differentiation-related proteins. METTL8 and JNK signaling intermediate factor Mapkbp1 interact with mRNA to inhibit translation, affecting the differentiation of mESCs but not their pluripotency [148].

METTL1 plays an important role in maintaining and promoting the differentiation function of undifferentiated cells within the METTL family. The loss of METTL16 results in the instability of the hematopoietic stem cells and progenitor cells (HSPCs) transcription factor MYBL2b in vertebrates, causing HSPCs to be blocked in the G1/S cell cycle, and impairing cell proliferation capacity [149]. METTL16 knockout in mESCs will change the ribosome occupancy and RNA levels, leading to impaired pluripotency [130]. Knocking down METTL4 will inhibit stem cell differentiation into mature adipocytes [72]. In summary, the METTL family plays varying roles in the differentiation of different cell types but is important for maintaining cellular stability. Finding commonalities in similar molecules may lead to stable organ structure and function and may correct errors in cell differentiation caused by changes in the body's environment.

#### METTL family and mitochondria translation

In mammals, mitochondria are semi-autonomous organelles composed of nuclear DNA and proteins encoded by mitochondrial DNA (mt-DNA). With more than 1000 proteins encoded by nuclear DNA being localized in the mitochondria, and 13 proteins encoded by mt-DNA making up the essential components of the electron transport chain (ETC), they are involved in oxidative phosphorylation to generate ATP. Studies have revealed an enrichment of 6mA modifications in mt-DNA, with levels 1300 times higher than total DNA, especially under specific stress conditions. In vitro experiments have shown that 6mA significantly diminishes the transcriptional activity of the mitochondrial transcription complex, resulting in reduced transcription in human mitochondria. Under low oxygen conditions, the level of 6mA in mt-DNA further increases compared to steady-state conditions, inhibiting the binding of mitochondrial transcription factor (TFAM) and TFAM-dependent DNA bending, demonstrating that 6mA serves as a regulated mark of mammalian mt-DNA. Being the first lysine methyltransferase discovered to be associated with mitochondria, METTL20 regulates the interaction between succinate dehydrogenase and ETF-β through trimethylation of lysine residues, thus controlling the oxidation of fatty acids and electron transfer within the mitochondria via ETF [135, 136, 158]. The METTL9 methyltransferase complex I enhances respiration [164], and the presence of monomethylated histidine in HxH peptides reduces the zinc binding affinity of peptides containing the HxH motif [165], thereby affecting mitochondrial respiration. Furthermore, iron-sulfur (Fe-S) clusters serve as essential cofactors, primarily mediating electron transfer within the mitochondrial respiratory chain. Overexpression of METTL17 can restore the mitochondrial translation and bioenergetic defects in FNX (mitochondrial iron-sulfur (Fe-S) gene cluster biogenesis co-chaperone) deficient cells, as METTL17, with previously unidentified [Fe_4_S_4_]2 + cluster, binds to the mitochondrial ribosome in the late assembly process [117]. Knockdown of METTL15 leads to impaired mRNA translation and decreased synthesis of respiratory chain proteins in mitochondria, ultimately inhibiting mitochondrial respiration. In addition to regulating mt-mRNA, the METTL family methylates other mt-RNAs involved in the composition of mitochondrial ribosomes. METTL8 mediates the m3C modification on position 32 of mt-tRNA, affecting mitochondrial translation [121]. The loss of METTL17 affects the stability of 12S mt-rRNA and its associated proteins, and METTL17 regulates mitochondrial ribosomal function in a SAM-dependent manner [157]. Furthermore, the METTL family also has an impact on oxidative phosphorylation. The SAM-dependent methyltransferase METTL12 inhibits CS Lys-395 through methylation of citrate to regulate the citric acid cycle [134].

### METTL family and others

GAB1 and METTL13 co-localize in auditory sensory neurons, and METTL13 co-immunoprecipitates with GAB1 and SPRY2. Missense mutations in METTL13 can prevent the deafness caused by GAB1 gene mutations [161]. Genetic variations in METTL13 are associated with an increased susceptibility to postpartum emotional syndrome [162]. GWAS has revealed an association between non-synonymous genetic polymorphisms of the METTL4 gene and sensory impairment, contributing to the maintenance of human sensory perception [166]. Additionally, METTL16 is involved in promoting learning and memory function in mammals by stabilizing MAT2A mRNA [167]. Although these members of the METTL family are known to play a role in maintaining the body's external perception, the specific mechanisms behind their actions remain unclear. Furthermore, the m6A methylation modification mediated by METTL4 on Col17a1, Itgβ4, and Itgα6 inhibits translation, thereby maintaining epidermal homeostasis [160]. Moreover, METTL3 activates autophagosome formation, inhibits vascular smooth muscle cell proliferation, and restricts neointima formation after vascular injury by reducing the levels of phosphorylated mammalian target of rapamycin (p-mTOR) and cyclin-dependent kinase 1 (p-CDK1/CDC2) [159]. Additionally, METTL13 interacts with Ezrin, Rab8a, and Septin 2, reducing the number of ciliated cells with primary cilia in MA-10 interstitial cells, downregulating AMPK activity, and enhancing INSL3 expression, thus supporting in vivo abdominal testicular migration [163]. It is essential to note that the METTL family plays a crucial role in maintaining various functions in the human body. However, there is relatively less research focusing on their normal functions compared to their link with diseases. It is crucial to explore their key roles in normal human functions and their potential as diagnostic or dynamic monitoring markers for disease treatment outcomes, as well as for assessing body status.

### Dysregulation of METTL family in diseases

**Fig. 5:**
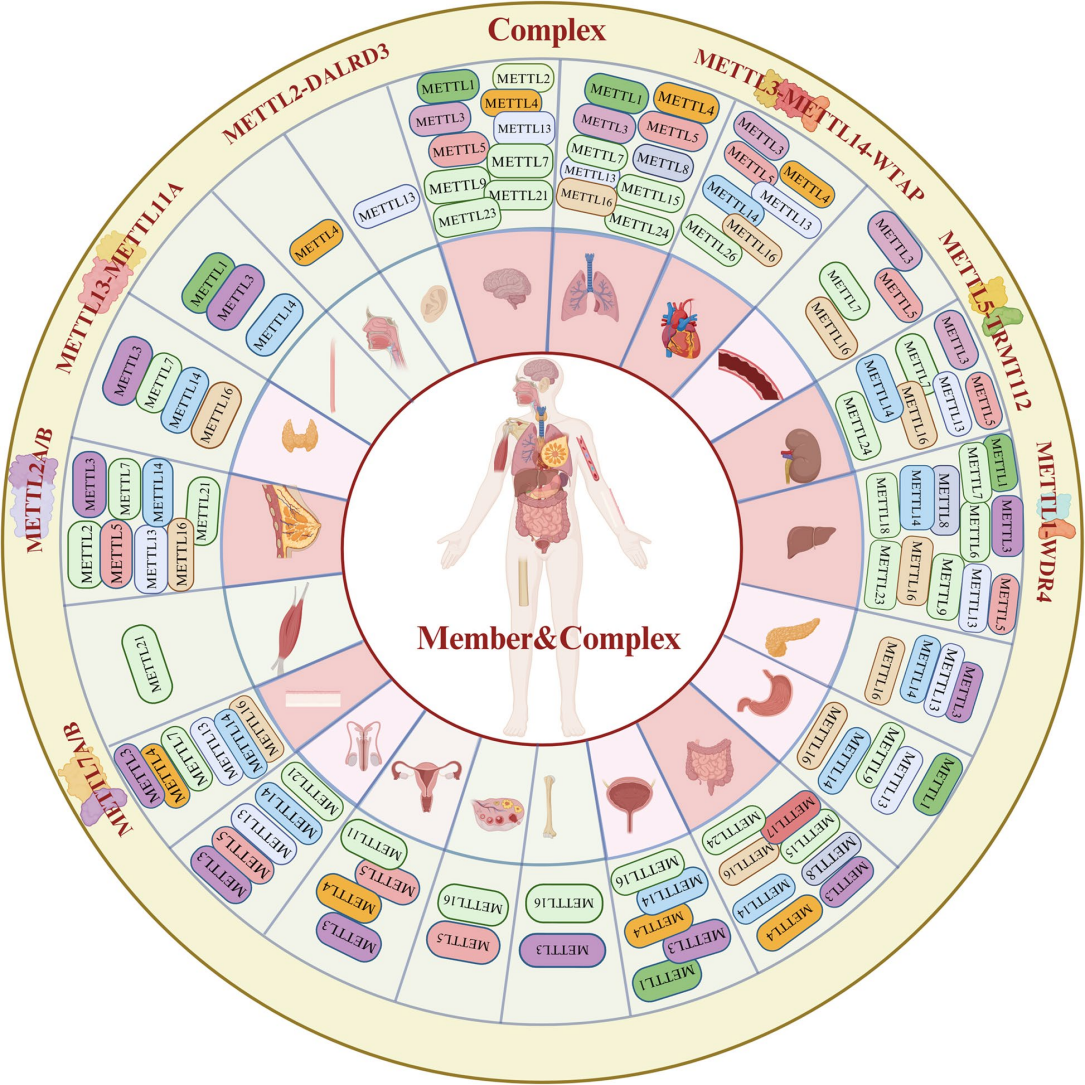
Distribution of the METTL family members in the human body. In recent years, research on METTL family members has focused on various organs. The studies have been more prevalent in the nerve, heart, lungs, liver, bowel, kidney, and breast, while comparatively less attention has been given to muscle, ears, mouth, ovary, and testis. Also exemplifies the intricacies of the situation

In addition to investigating the cellular or organismal functions of the METTL family, there is a heightened emphasis on disease research (Fig. 5). The dysregulation of METTL family members has been observed in a range of conditions including cancer, neurological disorders, and cardiovascular diseases (Table 2). Several research findings suggest that the methylation modification by the METTL family influences the onset and progression of diseases. As research progresses, the potential of the METTL family as crucial diagnostic and therapeutic markers is boundless.

**Table 2:** Dysregulation of METTL Family in Diseases without Cancer

	**Regulators**	**Targets**	**function**	**References**
**Nervous System**	METTL1	RNA(m7G)	METTL1-WDR4 methyltransferase complex and the genetic predisposition of glioma	[27]
		–	model the neurodevelopmental Rubinstein Taybi Syndrome caused by mutations in the genes encoding CBP/p300 acetyltransferases	[28]
		Sptbn2 (m6A)	METTL1-WDR4 and hippocampal neurogenesis and spatial memory	[31]
		–	neuroblastoma susceptibility	[168]
	METTL2	DALRD3 tRNA(m3C)	exhibit developmental delay and early-onset epileptic encephalopathy	[169]
	METTL4	–	variants and genes implicated in autosomal recessive and X linked ID	[170]
	METTL5	rRNA(m6A)	reduced body size and metabolic defects	[83]
		rRNA(m6A)	maintain brain function and intelligence	[171]
		–	associate with ID	[172]
		RNA	m6A modification enriched in transcripts associated with psychiatric disorders including conditions with clear cognitive deficits	[173]
		S6	improve the efficiency of nuclear transfer cloning	[174]
	METTL7	–	RNA editing	[175]
	METTL14	–	the significant factor for DFS and OS in NB patients	[176]
	METTL16	MAT2A(m6A)	improve hippocampal global m(6)A levels, plasticity of dendritic spine, learning and memory	[167]
		MAT2A(m6A)	facilitate early development	[177]
		–	the significant factor for DFS and OS in NB patients	[176]
**Cardiovascular Disease**	METTL1	TMEM11	cardiomyocyte proliferation	[24]
		RNA(m7G)	a novel pro-fibrosis role	[25]
	METTL2	–	a relationship with hypertension	[178]
	METTL3	RNA(m6A)	cardiomyocytes differentiation	[179]
	METTL4	–	associate with HFpEF	[180]
		–	affect mRNA stability, regulate transcription, and modulate pre-mRNA splicing	[181]
	METTL5	SUZ12(m6A)	cardiac hypertrophy through m6A modification	[182]
		RNA(m6A)	modulate AS progression by regulating m6A methylation levels	[183]
	METTL13	c-Cbl (Lys methylation)	regulate cardiac contractile function and remodeling	[184]
	METTL14	RNA(m6A)	cardiomyocytes differentiation	[179]
	METTL16	–	the methylation status of the 3'UTR region of METTL16 subsequently affecting its transcriptional activity in SCD-CAD	[185]
		RNA(m6A)	cardiomyocytes differentiation	[179]
**Others**	METTL3	YAP1(m6A)	promote melanin production	[186]
	METTL3	IL17A (m6A)	a significant pro-inflammatory factor in psoriasis	[187]
	METTL16	DNA-repair-related genes(m6A)	essential regulatory machinery to maintain genome integrity and erythropoiesis	[188]
	METTL15	–	upregulation Diabetic nephropathy	[189]
	METTL23	–	relate to Normal tension glaucoma	[190]
	METTL16	CIDEA(m6A)	associate with non-alcoholic fatty liver disease	[191]
	METTL16	–	the establishment of implantation and maintenance of pregnancy	[192]
	METTL3	RNA(m6A)	female fertility	[193]

#### Cancer

**Fig. 6:**
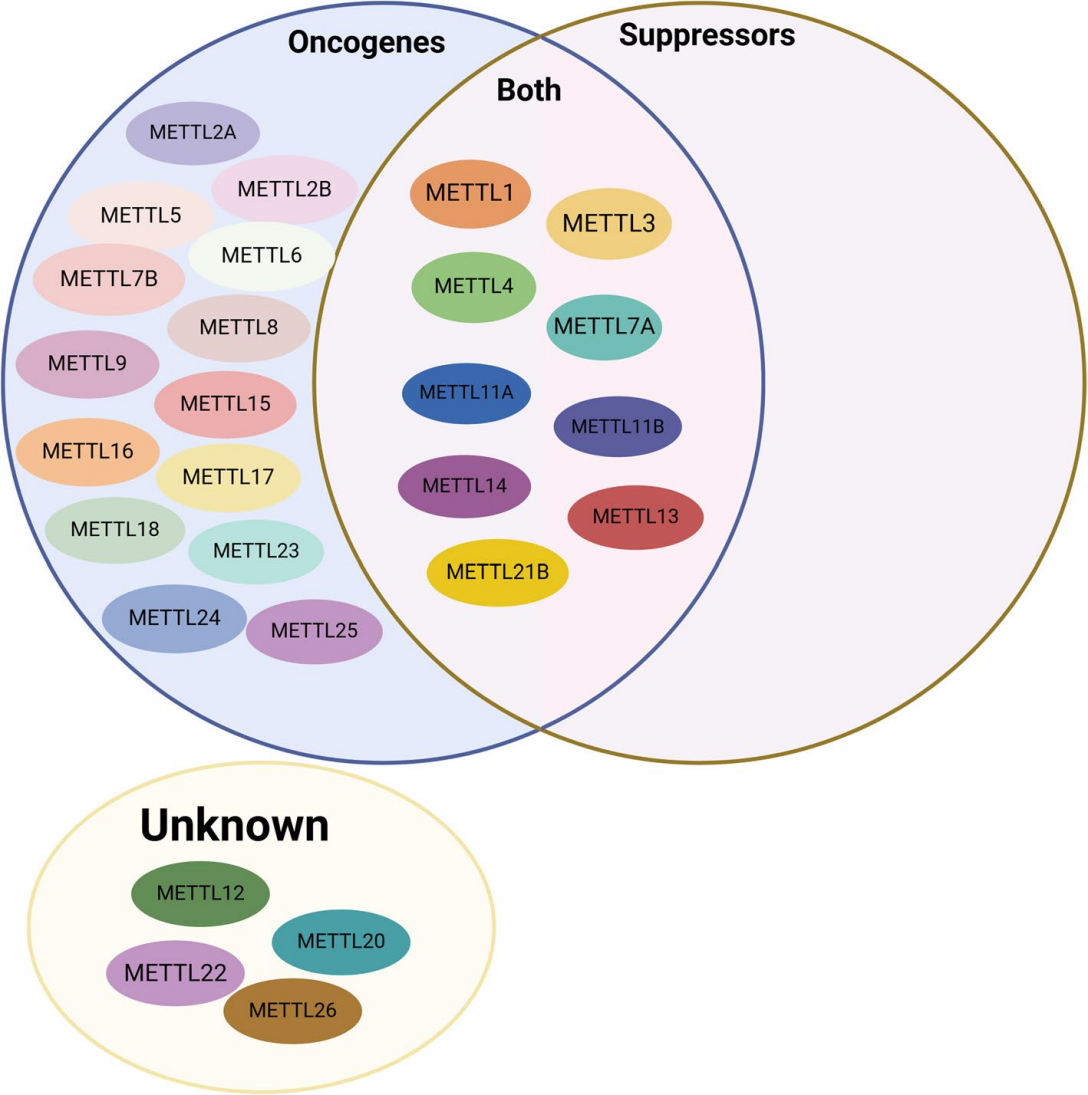
Summary of the Known Roles of the METTL Family. As of now, the majority of METTL proteins have demonstrated oncogenic activity, with some functioning as both oncogenes and tumor suppressors, but none have shown exclusive tumor suppressor activity

In the context of cancer, mutations in the METTL family can lead to malignant cell proliferation, culminating in cells with invasive and metastatic properties, thus contributing to the initiation and progression of cancer. Various members of the METTL family demonstrate either pro-cancer or anti-cancer effects within different types of cancer tissues (Fig. 6). For example, METTL16 enhances mRNA translation efficiency, thereby influencing the self-renewal of hepatic cancer stem cells and impacting the development of liver cancer [150]. Furthermore, elevated copper levels induce lactosylation of non-histone METTL16-K229, resulting in increased m6A levels on FDX1 mRNA and subsequent copper-induced cell death in gastric cancer cells [194]. On the other hand, the reduction of METTL1 leads to a substantial decrease in m7G modification levels on tRNAs, consequently destabilizing tRNAs and contributing to the onset of AML [15]. Additionally, the METTL5-mediated m6A modification of 18S rRNA promotes the translation of oncogenic mRNAs, thereby facilitating the progression of intrahepatic cholangiocarcinoma [79]. It is noteworthy that the METTL family members can modulate translation through the formation of complexes and double modifications, ultimately contributing to cancer development. For instance, the dual m6A/m7G methylation modification mediated by METTL3/METTL1 enhances TROP2 translation, thereby promoting the development of bladder cancer [140]. Moreover, the METTL family is involved in the regulation of iron-mediated cell death in tumors, with various specific mechanisms yet to be fully elucidated [194].

This includes the targeting of FTH1 and pre-miR-26a by METTL1, leading to increased RNA m7G levels and promoting miRNA maturation, ultimately resulting in iron-mediated cell death [18]. Furthermore, the involvement of the miR-30c-5p targeting the METTL3/KRAS axis is crucial in driving the progression of cervical cancer, shedding light on the molecular mechanisms underlying cervical cancer growth and metastasis [195]. Additionally, IGF2BP2 regulates TFRC mRNA methylation via METTL4, thereby playing a role in the modulation of iron-mediated cell death and the promotion of colorectal cancer growth [196]. Tumor cell iron death can induce an immune response, promoting infiltration and activation of the B cell population [197]. It is important to further explore the mechanisms by which the METTL family induces iron-mediated cell death via an immune response, as this understanding will be crucial for future strategies aimed at inducing tumor cell death through various treatment methods.

In hepatocellular carcinoma, a positive feedback loop involving METTL13, eEF1A, and HN1L has been identified. Within this loop, HN1L activates AP-2γ, thereby promoting its own co-activation with TCF3 and ZEB1, both regulated by METTL13 [109]. Meanwhile, eEF1A, a highly conserved and fundamental non-ribosomal component within the ribosome complex, participates in the translocation process during ribosomal translation and is one of the most abundant proteins in eukaryotic cells [198]. Additionally, METTL16 forms a complex with eIF3a/b to initiate mRNA translation. In breast cancer and leukemia cells, eIF3 facilitates the translation of METTL3, a process that is inhibited by 5'-end m6A mutations or deletions. Furthermore, phosphorylation-altered eIF2α decreases METTL3 levels, thereby impacting chemotherapy survival rates and immune cell migration [199]. Although the METTL family exhibits feedback interaction with translation factors, the exact mechanisms involved require further exploration, which would provide a crucial foundation for the study of related drugs.

#### METTL Family in the nervous system

The METTL5 protein, enriched in hippocampal neurons and synapses, is expressed in various substructures of the brains of rodents and humans [171]. Mutations in the METTL family result in the inability to maintain neural function. Knocking down METTL5 in a mouse model leads to a decrease in learning ability [200]. Genetic variation in the METTL5 gene with the help of TRMT112 [83] results in craniofacial and neurological developmental abnormalities, as well as intellectual disability syndrome (ID) with microcephaly [201]. METTL16 promotes MAT2A expression by regulating the m6A level at the 3' end of MAT2A mRNA, increasing its stability, and participating in the improvement of overall m6A levels, dendritic spine plasticity, learning, and memory in the hippocampus [177]. Spatial memory is also regulated by METTL3 [202]. The METTL family may be an important molecular component for human perception of external environmental changes. Genetic defects in METTL1 cause impaired hippocampal neurogenesis and spatial memory in adult mice [31]. Mutations in METTL family members lead to various types of neurological disorders. METTL1 gene polymorphism is associated with neuroblastoma and Rubinstein-Taybi syndrome (RSTS) [27, 28, 168]. METTL13 and METTL22 have been found to be associated with depression [162, 203]. After traumatic brain injury in mice, neurofunctional impairments, cognitive impairments, and neuronal apoptosis are reduced along with decreased CirMETTL9 levels [204]. Adult hippocampal neurogenesis in AD is associated with mRNA m7G methylation [205]. Elevated RNA m6A levels induced by various risk factors increase the probability of developing AD [206]. Research is needed to further understand the nature of the methylation modifications caused by the METTL family for maintaining neural function [207]. Balancing the expression of the METTL family and its methylation activity may be used to correct neurological disorders and is one of the directions for future research in the treatment of related nervous system diseases.

#### METTL family and cardiovascular disease

In aging male animals, METTL3 is downregulated in the aorta and atria, but remains upregulated in tissue from patients with heart failure accompanied by preserved ejection fraction (HFpEF) [180]. Studies indicate that METTL13 is capable of reversing heart failure caused by hypoxia or infarction in myocardial cells, primarily by reducing SERCA2a degradation through decreased ubiquitination [184]. Although METTL3 exerts a protective effect, its elevated levels may also have deleterious effects on the cardiovascular system. In a rat model of hypoxia-inducted pulmonary arterial hypertension and in hypoxia-induced or PDGF-BB-induced PASMCs, both METTL3 and YTHDF1 are upregulated [208]. METTL3 contributes to the upregulation of eIF2α mRNA methylation modification, leading to the proliferation of pulmonary artery smooth muscle cells (PASMCs) in MCT-PAH rats, resulting in pulmonary arterial hypertension (PAH) [143]. Besides the impact of METTL3 expression and methylation modification on the cardiovascular system, other members of the METTL family are also involved in maintaining and regulating cardiovascular function. In myocardial cells, TMEM11 interacts directly with METTL1, boosting the level of ATF5 mRNA m7G modification and promoting the transcription of the cyclin-dependent kinase inhibitor Inca1. Consequently, this weakens the action of cyclin A1 and leads to the inhibition of myocardial cell proliferation [24]. Furthermore, METTL16 regulates inflammation and apoptosis-related pathways involved in lipopolysaccharide (LPS)-induced myocardial injury [209]. Increased levels of RNA m7G modification mediated by METTL1 were observed in cardiac fibrosis tissue and TGF-β1-induced cardiac fibroblast proliferation, and knocking out METTL1 in fibroblasts alleviated heart failure and cardiac fibrosis induced by myocardial infarction [25]. METTL5 affects translation by regulating the m6A levels on SUZ12 mRNA, thereby reversing the inhibition of the myocardial hypertrophy transcriptome [182]. Sudden cardiac death (SCD) is associated with the 3' end methylation level of METTL16 [185].

#### Others

UVB radiation stimulates the overall m6A methylation modification in mesenchymal stem cells (MCs) and can elevate the expression of METTL3. This, in turn, increases the expression level of YAP1 through m6A modification, thereby activating the co-transcription factor TEAD1 to promote melanin production, resulting in skin pigmentation [186]. Elevated expression of METTL3 can lead to an increase in m6A modification of IL17A mRNA, a significant pro-inflammatory factor in psoriasis, promoting the onset of the condition [187]. Furthermore, the METTL family is implicated in various diseases including erythrocyte differentiation [188], diabetic nephropathy [189], respiratory distress syndrome [210], glaucoma [190], non-alcoholic fatty liver disease [191], cryptorchidism, pregnancy-related issues, infertility [192, 193], and intervertebral disc herniation.

## Therapeutic potential and future directions

While there have been many studies on small molecule inhibitors targeting m6A modification, the current focus of research on the METTL family has led to the development of numerous small molecule inhibitors targeting METTL3 or the METTL3/14 complex, with relatively few investigations focused on small molecule inhibitors targeting other members of the METTL protein family [211]. Some small molecule inhibitors of METTL3/14 are currently in preclinical and clinical development, but their specific outcomes remain unknown [124].

There have been relatively few investigations into the relationship between the METTL family and radiotherapy and chemotherapy. Some have speculated on this relationship through computer analysis but have not conducted in-depth research [212, 213]. Some members of the METTL family have been associated with resistance to chemotherapy drugs such as cisplatin [214], docetaxel [214], doxorubicin [34], anlotinib [14], methotrexate [215], and paclitaxel [216], but their mechanisms of resistance have been limited by experimental research. In methotrexate-resistant cell lines, increased expression of METTL7A and DDP4 activates the PI3K/AKT, ERK1/2, and STAT3 pathways, inhibiting apoptosis in choriocarcinoma cells, thus promoting methotrexate resistance [215]. Summarizing existing research results, the METTL family can influence the sensitivity of radiotherapy and chemotherapy through its methylation activity. METTL16, by binding to the m6A site of the 3' end of prostate transmembrane protein androgen-induced protein 1 (PMEPA1), reduces its mRNA stability, thus inhibiting the proliferation of bladder cancer cells and increasing sensitivity to cisplatin, mediated by the autophagy pathway [217]. Complexes are also involved. In animal experiments, METTL1/WDR4-mediated m7G tRNA modification is a key factor in promoting resistance to Lenvatinib [34]. Under conditions of sublethal heat stress induced by radiofrequency ablation (IRFA), the enhanced translation of SLUG/SNAIL is due to METTL1 and tRNAm7G modification, which inhibits the malignant potential of hepatocellular carcinoma cells [218] and can block the METTL1-TGF-β2-PMN-MDSC axis, promoting anti-tumor immunity and preventing HCC recurrence [219]. Radiotherapy and chemotherapy are effective means of cancer treatment, and increasing patients' sensitivity to them could effectively kill cancer cells. Due to the large number of members in the METTL family, investigating the commonalities in METTL family members that induce resistance to radiotherapy and chemotherapy can be a direction for future drug development in radiotherapy and chemotherapy.

Clinical treatment now emphasizes "precision treatment", with limited use of chemotherapy in patient treatment. Thus, advocating combination therapy, including Programmed death-1 (PD-1), has demonstrated improved anti-tumor efficacy and higher remission rates [220]. PD-1 is a cell surface receptor that plays a pivotal role in regulating T cell exhaustion as a T cell checkpoint and is closely associated with immunotherapy. METTL16 reduces the stability of PD-L1 mRNA via its m6A modification, consequently increasing the number of tumor-infiltrating immune cells in the immune microenvironment of cervical cancer, such as plasma cells and regulatory T cells [221]. The METTL family not only regulates PD-1 expression, but emerging studies suggest its role in modulating sensitivity to anti-PD-1 treatment. However, due to individual differences and treatment limitations, novel treatment strategies and combined treatment plans are required for effective anti-PD-1 immunotherapy. METTL3 and METTL14 inhibit mRNA m6A modification, enhancing the response of colorectal cancer and melanoma with good mismatch repair or low microsatellite instability (pMMR-MSI-L) to anti-PD-1 treatment while increasing the infiltration of CD8 + T cells and IFN-γ, Cxcl9, and Cxcl10 in tumor-infiltrating cells [222]. The efficacy of anti-PD-1 is linked to m6A modification, and upregulation or inhibition of members of the METTL family significantly impact the level of cell methylation. The potential of combined immunotherapy requires further exploration. Overexpression of METTL16 in CRC cells results in a decrease in the proportion of PD-1 positive T cells, synergistically affecting the growth of CRC cells in vivo [223]. In a preclinical mouse model of intrahepatic cholangiocarcinoma (ICC), PD-1 treatment simultaneously blocked METTL1 and its downstream chemokine pathway (CXCL8 in the human body and CXCL5 in the mouse body), boosting the immune response in the mice [224]. Studies have explored the combination therapy of dual immune checkpoint inhibitors (ICI) [225]. These inhibitors have novel mechanisms of action and/or therapeutic applications that can achieve effects unattainable with single antibodies. By the end of 2023, 14 dual immune checkpoint inhibitors have been approved: 11 for cancer treatment and 3 for non-tumor indications. Dual immune checkpoint inhibitors exist in different forms, targeting different entities, and exert anti-cancer effects through distinct molecular mechanisms [226]. The combination of the parbocizumab antibody, trastuzumab, and first-line chemotherapy substantially extended the progression-free survival in patients with metastatic HER2-positive gastric or esophageal cancer, particularly in those with a tumor PD-L1 composite positive score greater than or equal to 1 [227]. Anti-CTLA4 and anti-PD-1 exacerbate autoimmune colitis, activate inflammatory responses, and promote infiltration of myeloid cells, macrophages, dendritic cells, monocytes, and neutrophils, amplifying the body's immune response and enhancing treatment effects [228]. Reliable immune therapy targets will significantly enhance the efficacy of immune therapy, with the METTL family representing such a molecule. The METTL family is associated with the immune response [124], yet there are relatively few studies on its application in immunotherapy. The expression and methylation modification of the METTL family could be developed to target METTL family members' MTase structure domain for the creation of anti-METTL family antibodies, paving the way for a bidirectional treatment of METTL family mutations related diseases, and this could become a direction for future exploration.

Parallel to specific 3' end mRNA binding, miRNA primarily regulates the expression of target genes through two mechanisms. This includes complete complementary binding to degrade the target mRNA and imperfect binding to silence the gene post-transcription [229]. The role of miRNA in cancer diagnosis and treatment is gaining significant traction, especially in exploring its therapeutic effects through the specific binding of miRNA to mRNA sequences [230]. Liposomes, initially reported as drug carriers in the late 1970s, have since garnered extensive research interest [231]. In the first clinical trial of miRNA-based tumor intervention, liposomes carrying miR-34a mimic, known as MRX34, were administered to patients with inoperable liver cancer and other solid malignant tumors [232]. This study marked the first demonstration of the potential for liposome-mediated miRNA delivery. Advancement in technology has led to the use of lifeless bacterial minicells nanoparticles as carriers to deliver miR-16 for inhibiting the expression of intracellular oncogenes in the treatment of patients with recurrent malignant pleural mesothelioma [233, 234]. miRNA regulates the mRNA of METTL family members in various ways. For instance, the 3' end of METTL7A binds to miR-6807-5p, leading to the silencing of METTL7A and inhibiting odontogenic differentiation in dental pulp stem cells (DPSCs) [87, 235]. Meanwhile, studies have demonstrated that miR-30c-5p promotes iron death of cervical cancer cells by inhibiting the METTL3/KRAS axis [195], consequently inhibiting tumor growth, migration, and metastasis. Inconsistencies between the levels of METTL13 protein and its mRNA have been observed in lung cancer, breast cancer, and liver cell carcinoma, showing a negative correlation [104]. Notably, miR-16 binding to the 3' end of METTL13 inhibits its expression, hence regulating the EMT and NF-κB signaling pathways to suppress liver cancer cell growth [105]. RNA immunoprecipitation experiments on colorectal cancer have suggested that circ-METTL9, containing multiple exons, may serve as a miRNA sponge, interacting with miR-551b-5p [236]. Application of drug delivery systems to convey miRNA to locations with high concentrations of METTL family member mRNA can restrain the oncogenic effects of these members. Another approach involves modifying the structure of METTL family members to augment their affinity with target molecules, thereby enhancing their binding ability with circRNA. These drug-carrying nanoparticles can transport not only miRNA but also antibodies for relevant overexpressed genes, such as anti-METTL antibodies or immune-targeted drugs(such as PD-1), to increase the effectiveness of the drug delivery system [233]. Using this system to deliver miRNA into the body to inhibit the expression of the METTL family holds promise, given the improvement in drug targeting, circulation time, and efficacy brought about by drug delivery systems [237]. Additionally, intracellular miRNA enhances immune function; for instance, extracellular vesicle miRNA-16-5p from M1 type macrophages can boost T cell-dependent immune response in gastric cancer patients by regulating PD-L1 [238]. Hence, the potential application of nanotechnology and miRNA for METTL family-related cancer treatment appears promising.

## Discussion

**Fig. 7:**
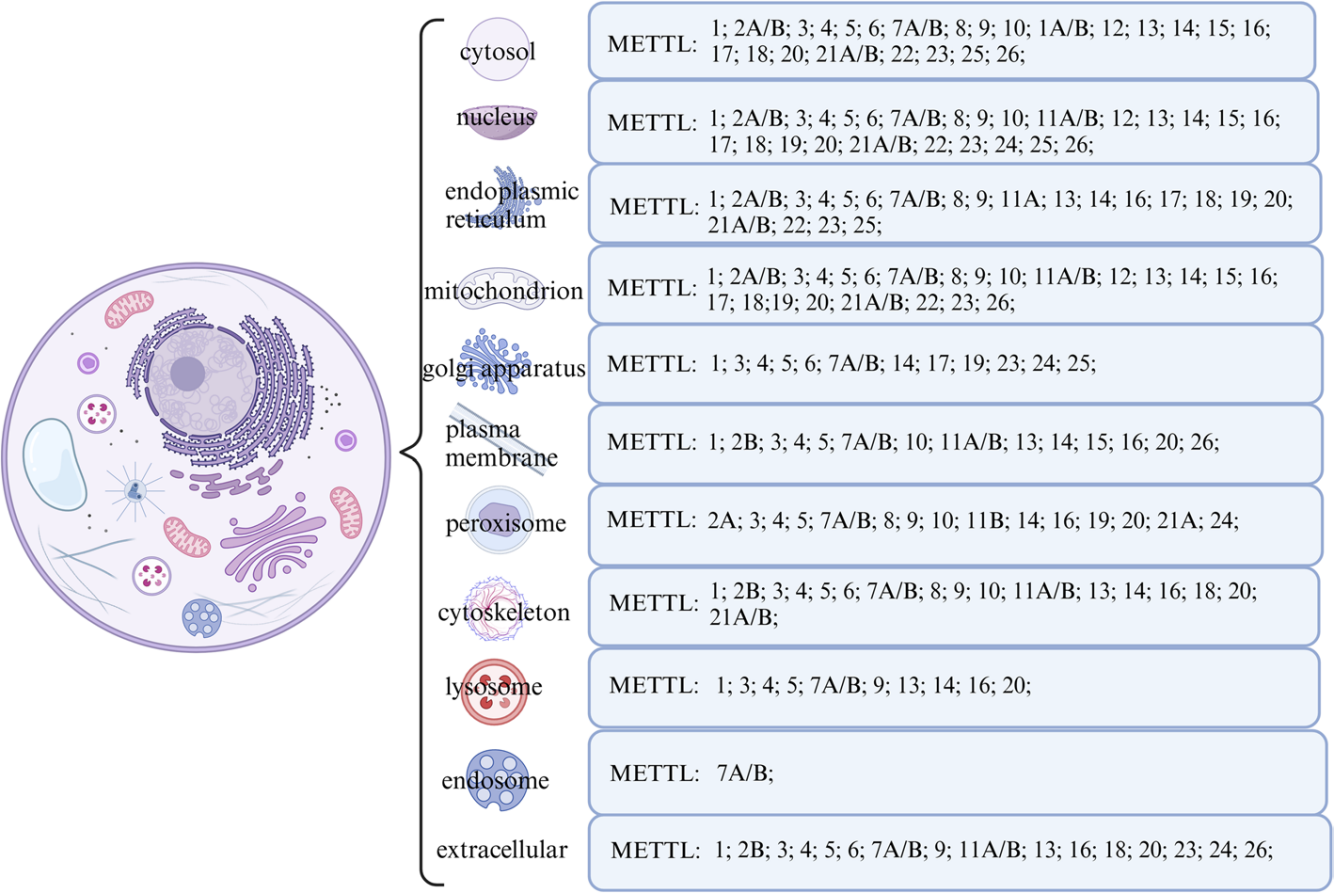
Distribution of the METTL Family in Cells. The current research has already elucidated the locations where the members of the METTL family are found in the cell based on data obtained from Genes Card Database

The METTL family is involved in regulating the methylation modification of tRNA, rRNA, and mRNA in both the cytoplasm and mitochondria, affecting RNA stability and translation function. Moreover, the METTL family exerts control over translation through the regulation of translation factors. While current research primarily focuses on understanding the interaction between METTL family members and RNA that affects translation, limited research has been conducted on the interaction between translation factors and the METTL family. Eukaryotic translation encompasses initiation, elongation, and termination processes, with the elongation of the peptide chain requiring the involvement of eEF factors. Specifically, within the ribosome, eEF1A binds to GTP, activating it to promote the translocation of aminoacyl-tRNA from the P site to the A site, and additionally, establishing an indirect link between the cellular cytoskeleton and translation. Studies have demonstrated the widespread presence of lysine methylation on eEF1A in various organisms [239], with lysine residues on eEF1A being regulated by the methylation modification of METTL family members such as METTL13, METTL21, and METTL10. The METTL family, belonging to the 7BS methyltransferase subfamily, has been associated with methylating K36 on eEF1A, thereby enhancing translational dynamics and accelerating the translation speed of different codons [240]. METTL13 plays a highly specific role in methylating the N-terminus and Lys55 of eEF1A, promoting the synthesis of specific amino acids, a process prevalent in mammalian cells and tissues. Furthermore, members of the METTL family also regulate eIF factors, such as METTL16, which specifically binds to eIF3a/b in the cytoplasm, exerting an influence on translation. Notably, certain members of the METTL family are present on the cellular cytoskeleton (Fig. 7), given the association of eEF1A with this structure. Investigating the localization of METTL family members and translation factors at different stages of cell division and their respective binding and reaction sites within the cell holds potential for future exploration.

The METTL family plays a vital role in translation by regulating transcription and influencing the abundance of RNA within the cell. Translation factors play a role in protein synthesis. Studies have demonstrated that METTL13 promotes translation by methylating eEF1A. In liver cancer, the METTL13-eEF1A-HN1L complex forms a positive feedback loop. Furthermore, the METTL family not only governs the transcription and translation of specific mitochondrial protein genes but also affects the reduction of energy production associated with respiratory chain proteins. Both the METTL family and translation factors are crucial molecules within the cell, and certain members may have mutual promotion effects. If a combined treatment targeting the METTL family and translation factors with immune checkpoint inhibitors (ICI) is implemented, it can not only reduce the indirect energy production within the cell, leading to a decrease in the energy supply to the translation process, but it can also directly inhibit translation.

Methylation is a common molecular marker that is impacted by both internal and external environmental changes, affecting the overall methylation level of cells. Thus, detecting or monitoring changes in methylation levels holds potential as an important diagnostic and prognostic tool for future disease management. Various human samples, including tissues, blood, urine, serous cavity effusion, lavage fluid, feces, and swabs, are routinely collected for assessing methylation levels in different tumors. The predictive and responsive nature of DNA methylation modification provide valuable insights for timely patient diagnosis and treatment. In a study of 103 colonoscopy-diagnosed colorectal cancer patients, ColonSecure testing demonstrated superior detection efficiency of methylation levels compared to CEA, CRP, and CA19-9 in 89 patients [241]. Moreover, high methylation sites associated with the NOTCH1/RUNX3/epidermal growth factor receptor signaling pathway exhibited elevated methylation in cheek tissue carcinoma samples from smokers and in situ lesions of advanced lung cancer, predicting the development of lung cancer in cheek samples collected from smokers [242]. Various host cell methylated genes such as SOX1, PAX1, JAM3, EPB41L3, CADM1, and MAL have been investigated for predicting cervical lesions, with PAX1 gene methylation showing the most significant correlation with the progression of cervical intraepithelial neoplasia and cervical cancer occurrence. PAX1 gene methylation levels increase with the grading of cervical lesions, and new cell DNA methylation biomarkers such as SOX1 and ZNF582 continue to emerge [243]. Furthermore, RNA m6A methylation is widely present in diseased tissues and its detection, in addition to DNA methylation, may enhance the detection rate of precancerous lesions or cancer, thereby benefiting high-risk populations and patients. Notably, m6A-modified CENPK RNA exhibits high expression and is positively correlated with the overall and recurrence-free survival rates of cervical cancer patients [244]. While DNA methylation detection has begun to be used in clinical practice, there is a shared aim among scientists to develop effective, convenient, and affordable methods for this purpose. Continuous experimental screening is expected to lead to the discovery of the most efficient means for detecting different cancer types using a variety of samples or methods.

The occurrence and progression of diseases are associated with changes in related molecules. While numerous studies have demonstrated the association between disease pathways and molecules, the identification of crucial molecular targets for disease prevention, early diagnosis, and treatment remains elusive. In most pathological tissues, the levels of the METTL family are significantly increased compared to normal tissues, and the higher the degree of lesion, the higher the levels of the METTL family. Normal expression of certain members can maintain normal human functions; once altered, they can be detected in patient plasma, similar to common tumor markers, upon causing cancer. For instance, METTL13 from primary tumor cells can enter the circulation by releasing chemical substances that dissolve blood vessel walls. Members of the METTL family are widely distributed in the cytoplasm, mitochondria, and cell nucleus, and the detection of METTL levels in cell lysates might be a future direction for disease diagnosis. Detecting the levels of the METTL family in plasma and cell lysates may improve the detection rate of patients. Both the detection of tumor markers and circulating tumor cells are based on the patient's tumor burden. If drug treatment is effective or the patient's prognosis is good, the expression levels of the METTL family and the number of circulating tumor cells in blood are likely to decrease. Therefore, detecting the METTL family in plasma and tumor cells in blood can assess the therapeutic effect of the drugs and the prognosis.

Alterations in the METTL family levels serve as indicators of tumor occurrence and progression. Methylation modification has garnered escalating interest in its role in disease diagnosis, treatment, and prognosis in recent years. The translation process, integral to protein expression, necessitates not only translation factors but also a supply of energy. Certain METTL family members are linked with translation factors, however, the relationship between the METTL family and translation remains largely unexplored. The emergence of detection methods and treatment drugs for the METTL family is underway, it contains enormous potential. As a 7BS methyltransferase subfamily, the function of the METTL family necessitates ongoing research.

## Data Availability

Not applicable.
